# Enforced Clonality Confers a Fitness Advantage

**DOI:** 10.3389/fpls.2016.00002

**Published:** 2016-01-26

**Authors:** Jana Martínková, Jitka Klimešová

**Affiliations:** Department of Functional Ecology, Institute of Botany, Czech Academy of SciencesTřeboň, Czech Republic

**Keywords:** bud bank, disturbance, fitness, life strategy, ontogeny, ramet, root fragment, seed number

## Abstract

In largely clonal plants, splitting of a maternal plant into potentially independent plants (ramets) is usually spontaneous; however, such fragmentation also occurs in otherwise non-clonal species due to application of external force. This process might play an important yet largely overlooked role for otherwise non-clonal plants by providing a mechanism to regenerate after disturbance. Here, in a 5-year garden experiment on two short-lived, otherwise non-clonal species, *Barbarea vulgaris* and *Barbarea stricta*, we compared the fitness of plants fragmented by simulated disturbance (“enforced ramets”) both with plants that contemporaneously originate in seed and with individuals unscathed by the disturbance event. Because the ability to regrow from fragments is related to plant age and stored reserves, we compared the effects of disturbance applied during three different ontogenetic stages of the plants. In *B. vulgaris*, enforced ramet fitness was higher than the measured fitness values of both uninjured plants and plants established from seed after the disturbance. This advantage decreased with increasing plant age at the time of fragmentation. In *B. stricta*, enforced ramet fitness was lower than or similar to fitness of uninjured plants and plants grown from seed. Our results likely reflect the habitat preferences of the study species, as *B. vulgaris* occurs in anthropogenic, disturbed habitats where body fragmentation is more probable and enforced clonality thus more advantageous than in the more natural habitats preferred by *B. stricta*. Generalizing from our results, we see that increased fitness yielded by enforced clonality would confer an evolutionary advantage in the face of disturbance, especially in habitats where a seed bank has not been formed, e.g., during invasion or colonization. Our results thus imply that enforced clonality should be taken into account when studying population dynamics and life strategies of otherwise non-clonal species in disturbed habitats.

## Introduction

Clonality is defined as the production of new, genetically identical ramets with potential to become independent of their mother (Klimeš et al., 1997). It has been repeatedly documented that clonal growth brings benefits including resource acquisition, maternal support for new offspring, higher competitive abilities, independence from mates, and high ability of vegetative regeneration (Meloni et al., 2013; Wang et al., 2013; Fukui and Araki, 2014; Elgersma et al., 2015; Glover et al., 2015). Splitting of a clone into ramets is usually spontaneous and takes months to decades to be completed (Klimeš et al., 1997). However, it may be also suddenly realized by external force that fragments a plant (Wehsarg, 1954; Klimešová and Martínková, 2004). When such resulting plant fragments are capable of surviving and regenerating we term this process of fragmentation and subsequent regeneration “enforced clonality.” Although this process is not so essential for plants that otherwise split into ramets spontaneously, it might be crucial for otherwise non-clonal plants from disturbed habitats (Sosnová et al., 2014).

Enforced clonality relies upon fragmentation of the plant body. However, successful survival and growth subsequent to such severe intrusion on the plant's integrity is dependent on the ability of fragments to form missing tissues (Groff and Kaplan, 1988). Therefore, a root fragment may provide the foundation for a new ramet only when the root is able to form an adventive bud from which a shoot can emerge. Similarly, a shoot fragment must bear an axillary bud to continue growth and must also be able to produce adventive roots. A leaf fragment would need to form adventive buds that give rise to both shoot and roots to become a new individual. Although all types of successfully regenerating fragments can be observed in nature, those based on leaves are extremely rare (see examples for woody plants in Sagers, 1993 and for herbs in Klimešová and Klimeš, 2007). In aquatic conditions, plants use water as a substrate; thus, formation of roots is not necessary and body fragmentation is a common way of clonal growth in such habitat (Barrat-Segretain and Bornette, 2000; Boedeltje et al., 2003; Campbell, 2003). In terrestrial ecosystems, enforced clonality has been reported from arable land, with species displaying it receiving attention as weeds (Kefford and Caso, 1972; McIntyre, 1972; Klimešová et al., 2008). It has also been documented in ruderal habitats, where short-lived species survive severe disturbance and some new individuals establish from root fragments (Klimešová et al., 2008; Martínková et al., 2016). Enforced clonality, however, has importance in other contexts as well, including habitats with natural soil disturbances such as landslides, scree, and water erosion (Hess, 1909), colonization of new areas, spread of invasive plants (Bailey et al., 2009; Lin et al., 2012; Monty et al., 2015), and vegetatively propagated crops and ornamentals (Shepherd et al., 2013; de Souza et al., 2014; Birlanga et al., 2015).

We can summarize that according to empirical observations enforced clonality exists and has ecological importance. However, the evolutionary importance of enforced clonality would depend on whether, and to what extent, it confers a fitness advantage over alternative regeneration modes. Even though there are cases when enforced clonality is the only one way to regenerate and thus its advantage is not questionable (e.g., cultivated varieties of seedless crops, Roberts-Nkrumah, 2006, or naturally in *Armoracia rusticana* in Central Europe, Sampliner and Miller, 2009), after disturbance plants more generally regenerate from seed banks. Thus, to identify the evolutionary significance of enforced clonality it is necessary to determine whether there are situations in which ramets generated by enforced clonality have higher fitness than plants that emerge from the seed bank.

The regeneration of plant fragments is affected by external and internal factors as in any other plant vegetatively regenerating after injury. In addition to the ability to form missing tissues, to regenerate, a fragment must also have sufficient storage carbohydrates to provide energy and carbon for body renewal. Because carbohydrate storage fluctuates with phenology and ontogeny (Sosnová and Klimešová, 2009; Kaur et al., 2012; Bazot et al., 2013). Especially in short-lived monocarpic species, carbohydrate storage can negatively affect the success of vegetative regeneration since stored reserves are exhausted by generative reproduction and vegetative regeneration is thus limited (Klimešová et al., 2007; Martínková et al., 2008; Tolsma et al., 2010). Similarly, the ontogenetic phase of the mother plant at the time of fragmentation may affect the fitness of resulting ramets since regrowth of newly established ramets is probably stored reserves dependent. Nutrient availability and depth in the soil are other factors influencing fragment regeneration outcomes (Dietz et al., 2002; Li et al., 2013; Thomsen et al., 2013).

Regeneration from the seed bank after disturbance have also some limitations. Seed bank is not necessarily formed as in the case of species not forming seed bank (Fenner, 1995) bud also species have no seed bank at the new locality during colonization or invasion process (Gioria et al., 2012) or due to recurrent disturbance (Noble and Slatyer, 1980), low level of resources or lack of signals for flower initiation due to climatic conditions hindering successful seed production (Simpson et al., 1999). In the case when seed bank is formed, seeds can germinate only when conditions are suitable and signals for germination triggering are present (Bewley, 1997; Baskin and Baskin, 1998). Therefore, ecological and evolutionary significance of enforced clonality is related to seed bank and habitat attributes, but fundamentally to presence or absence of the seed bank.

To investigate whether enforced clonality confers an evolutionary advantage on injured individuals via increased fitness and to explore how fitness of enforced ramets is affected by ontogeny of the mother plant at the time of fragmentation, we established a 5-year garden experiment with *Barbarea vulgaris* and *Barbarea stricta*, two short-lived species with potential for enforced clonality. Whole-life seed production, whole-life viable seed production, ratio of viable seeds to all seeds and annual immediate reproduction served as a proxy of fitness. The experiment allowed us to address the following specific questions: (i) whether the fitness of enforced ramets is higher than the fitness of plants that regenerated from the seed bank at the time of disturbance; (ii) whether the fitness of enforced ramets is higher than the fitness of an unfragmented plant; and (iii) whether fitness of enforced ramets decreases with the ontogenetic phase of the mother plant at the time of fragmentation, i.e., decreases during generative reproduction of the mother plant at the time of fragmentation.

## Materials and methods

### Study species

*B. vulgaris* R. Br. and *B. stricta* ANDRZ. (Brassicaceae) are common European species. *B. vulgaris* occupy man-made, ruderal habitats (e.g., arable land, urban habitats, roadside ditches) that are subjected to frequent, severe anthropogenic disturbance, whereas *B. stricta* occurs in more natural habitats (i.e., pond banks, river alluvia, (Dvořák, 1992), that experience naturally occurring disturbance). Both are short-lived herbs typically reproducing once in their lifetime and behaving as biennials, but in certain conditions reproducing repeatedly and behaving as short-lived perennials (MacDonald and Cavers, 1991; Dvořák, 1992; Martínková et al., 2016). During the first year of life, these plants remain vegetative, with rosettes overwintering to the next growing season, when they form leafy flowering stalks. Both species are usually non-clonal, but enforced clonality has been reported from them (Martínková et al., 2008, 2016). In particular, after fragmentation of the root system, they are able to form adventitious buds on roots, successfully regrow and finish the reproductive cycle. *B. vulgaris* regenerates from roots more vigorously than *B. stricta* (Martínková et al., 2016). Both species form persistent seed banks and are able to germinate throughout the year (Hintikka, 1988; Baskin and Baskin, 1989; Martínková, pers. obs.).

### Experiment

Seeds for the experiment were collected during the year 2003 from South-Bohemian natural populations (15 populations for *B. vulgaris* and three for *B. stricta*). To minimize any effects of seed origin, for each of these species, the seed from all populations was mixed. During the winter, seeds were kept in dark, dry storage at room temperature.

#### Mother plants

In the spring of 2004, for each species, hundred 2.5 l containers were filled with a garden substrate-sand 2:3 mixture and five seeds were sown per container. The containers were placed outdoors in a random design in the experimental garden of the Institute of Botany in Třeboň, Czech Republic. One week after seedlings emergence, the number of seedlings was reduced to only one per container. Containers were then randomly assigned to four groups. Of these, three groups were set up to simulate establishment of enforced ramets originating from different ontogenetic phases of mother plants, number of replicates was 20 plants per group. In the first group, mothers were subjected to fragmentation during the first-year rosette phase (R1). The second group represented mothers subjected to fragmentation during the second-year rosette phase (R2). The third group represented mothers subjected to fragmentation during the reproductive phase (REP), number of replicates was 30. The fourth group comprised unfragmented plants (NO INJURY) and served to represent the scenario without disturbance. During the whole cultivation of all four groups, plants were regularly fertilized with NPK commercial solution without hormone addition and watered when necessary.

#### Enforced ramets—root fragmentation

**Figure 1:**
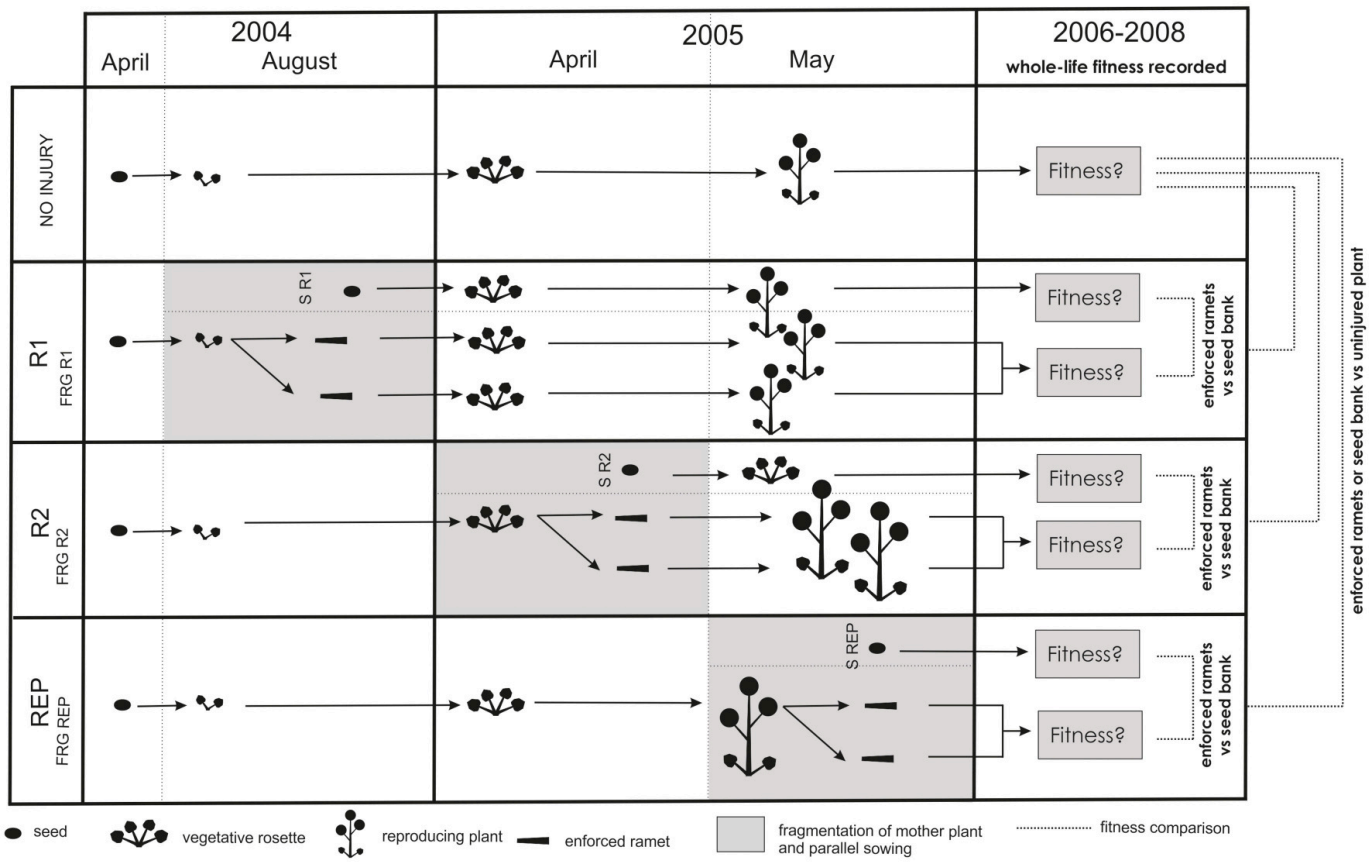
**Time and fitness comparison diagram of the experiment on ***Barbarea vulgaris*** and ***Barbarea stricta*****. Enforced ramets were established from mother plants in three ontogenetic stages. Parallel sowings were done at the same time as fragmentations of mother plants, to simulate regeneration from the seed bank after disturbance. FRG, enforced ramets; S, seed bank—parallel sowing; R1, seeds sowed or roots of mother plant fragmented in first-year rosette phase; R2, seeds sowed or roots of mother plant fragmented in second-year rosette phase; REP, seeds sowed or roots of mother plant fragmented in reproductive phase. NO INJURY, control unfragmented plants. Full descriptions of regeneration types and ontogenetic stages (and their abbreviations) are in Table 1.

Fragments were cut once in 2004 (fragmentation of R1 group) and twice in 2005 (fragmentation of R2 and REP groups), thus yielding three groups of enforced ramets: FRG R1, FRG R2, and FRG REP (Figure 1, Table 1). Each maternal plant served as the source of two root fragments, since we simulated a scenario in which severe disturbance establishes only two vegetative offspring from one mother to set the lowest level of possible multiplication. These two root fragments were each 6 cm long but differed in diameter and position in the root system. Six centimeters fragments were found to be able to successfully regenerate in both species (Martínková et al., 2016). Thus, the first fragment would be cut from the main root, specifically from the topmost part of the root directly under the hypocotyl. The second fragment would be cut from the first lateral root, directly behind its branching from the main root. Immediately after cutting, each fragment was placed horizontally into a 2.5 l container filled with a substrate:sand 2:3 mixture. Containers were placed in the experimental garden in a random design, regularly fertilized with NPK commercial solution without hormone addition and watered when necessary.

**Table 1:** **Overview of procedures used in experiment on ***Barbarea vulgaris*** and ***Barbarea stricta*** to yield different regeneration types as well as offspring from different mother plant ontogenetic stages**.

**Plant group**	**Fragmentation or sowing**	**Description**
**Mother plants**	**Sowing**	**Plants serving as source of enforced ramets**
R1	2nd of April 2004	Mothers, subjected to fragmentation in first-year rosette phase, of enforced ramets
R2	2nd of April 2004	Mothers, subjected to fragmentation in second-year rosette phase, of enforced ramets
REP	2nd of April 2004	Mothers, subjected to fragmentation in reproductive phase, of enforced ramets
**Enforced ramets**	**Fragmentation**	**Plants simulating enforced clonality**
FRG R1	18th of August 2004	Enforced ramets established from R1 mothers
FRG R2	6th of April 2005	Enforced ramets established from R1 mothers
FRG REP	20th of May 2005	Enforced ramets established from REP mothers
**Seed bank**	**Sowing**	**Plants simulating regeneration after disturbance from the seed bank**
S R1	18th of August 2004	Plants originating from seeds sowed concurrently with FRG R1
S R2	6th of April 2005	Plants originating from seeds sowed concurrently with FRG R2
S REP	20th of May 2005	Plants originated ing from seeds sowed concurrently with FRG REP
**Control**	**Sowing**	**Plants simulating conditions of no disturbance**
NO INJURY	2nd of April 2004	Plants of mother generation not subjected to fragmentation

#### Simulation of establishment from the seed bank

At the same time that enforced ramets were obtained, once in 2004 and twice in 2005, we did “parallel sowing,” i.e., sowing seeds at the same time that the fragmentation was done to the mothers of enforced ramets. This was done to simulate the situation in which regeneration after disturbance is possible only from the seed bank (Figure 1, Table 1), and would allow for comparison of plants that originated from mother plant fragmentation at various ontogenetic stages with plants that originated from seed approximately contemporaneously. Five seeds were sowed per 2.5 l container for each species separately in 20 replicates. One week after seedling emergence, the number of seedlings was reduced to only one per container. This yielded three groups of plants that originated from seeds: S R1, S R2, and S REP (Figure 1, Table 1). Containers were placed outdoors in the experimental garden, set in a random design, fertilized, and watered.

#### Seed collection and assessment of fitness variables

Seed collection and germination testing were done on all groups of plants every year of the experiment except the first, because both *Barbarea* species start reproducing in the second year of their lives. All seeds from each plant were trapped by wrapping reproducing plants in light white cloth when all their flowers had terminated flowering. The weight of all trapped seeds for each reproducing plant was evaluated after seed maturation. The average weight of one seed and the total number of seeds per plant were calculated from the weight of all trapped seeds and from the average weight of 30 seeds assessed from three replicates.

Germination tests were done every autumn following the reproductive season in standardized conditions of a chamber that followed a regime of 15 vs. 8 h and 23°C vs. 15°C for “day” vs. “night,” respectively. For each reproducing plant, 30 seeds in three replicates were placed on wet sand in Petri dishes. Over the course of the next 21 days, the number of germinated seeds would be recorded.

During in May, 2009, the experiment was terminated because the majority of plants had died out and the rest were so weak that high probability of death without reproduction was obvious.

For all plants, the following characteristics as a proxy of fitness were recorded: whole-life seed production (the sum of all seeds), whole-life viable seed production, ratio of number of viable to all seeds (VIABLE/ALL SEEDS) and annul immediate reproduction (ANNUAL REP). The numbers of all seeds and viable seeds were calculated as respective sums of the numbers of seeds produced by both enforced ramets of each mother plant. Annual immediate reproduction was calculated as an annual average, by first multiplying the mean number of viable seeds for each year and by that year's average seed weight, and next dividing this total by the number of reproductive years. VIABLE/ALL SEEDS and ANNUAL REP were both calculated as means of the both enforced ramets from each mother plant.

### Statistical analysis

As our data distributions do not fulfill the assumptions of traditional ANOVA (e.g., many of the plants do not produce any seed at all) we used analogous permutation tests in the program PERMANOVA+ for PRIMER (Anderson et al., 2008). Using these permutation tests, the pseudo-F ratio was calculated in a manner similar to the F ratio in traditional methods, but does not correspond to Fisher's F distribution, and the appropriate distribution which would be generated by a true null hypothesis is obtained by the permutation procedure (Anderson et al., 2008). The number of all seeds, the number of viable seeds, the ratio of viable/all seeds and annual immediate reproduction were treated as dependent variables in the analyses. Species affiliation, regeneration type, and ontogenetic phase of the mother plant were fixed factors. All tests were done for both species together in order to find general patterns between dependent variables and factors and were also done for each species separately to identify effects of regeneration type and ontogenic stage within individual species. We also performed permutation pairwise comparisons, which correspond to parametric *t*-tests (Anderson et al., 2008), to compare fitness characteristics among enforced ramets, parallel sowings, and uninjured plants.

## Results

### Number of all seeds

**Figure 2:**
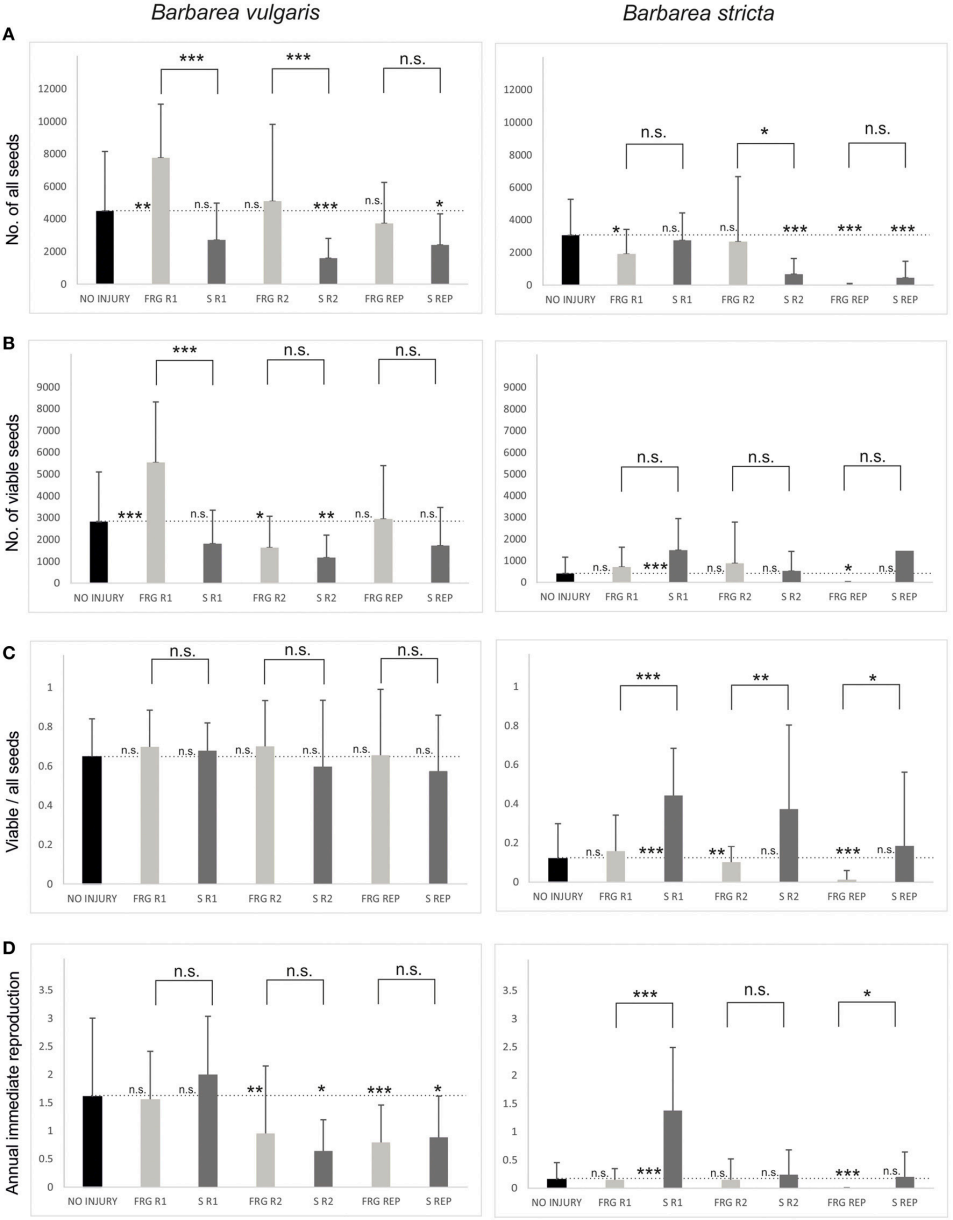
**Box diagrams of fitness characteristics obtained from experiment on ***Barbarea vulgaris*** and ***Barbarea stricta*** for individual regeneration types and ontogenetic stages in which the disturbance was inflicted**. **(A)** Number of all seeds; **(B)** Number of viable seeds; **(C)** Ratio viable/all seeds; **(D)** Annual immediate reproduction. FRG, enforced ramets; S, seed bank; R1, seeds sowed or roots of mother plant fragmented in first-year rosette phase; R2, seeds sowed or roots of mother plant fragmented in second-year rosette phase. REP, seeds sowed or roots of mother plant fragmented in reproductive phase. NO INJURY, control plants. Full descriptions of regeneration types and ontogenetic stages (and their abbreviations) are in Table 1. Means and 95% confidence intervals are plotted and significance range of pair-wise tests are shown. ^*^p < 0.05; ^**^p < 0.01, ^***^p < 0.00, n.s.—non-significant.

Whole-life seed production was significantly influenced by all tested factors and their interactions (Table 2). When looking at the species separately, in *B. vulgaris*, whole-life seed production of enforced ramets was higher than seed production of plants from parallel sowings if fragmentation occurred during the vegetative phase of mother plants, regardless of whether it was in the first or the second year of the plant's life (Table 3, Figure 2A). When comparing enforced ramets and uninjured plants, whole-life seed production of enforced ramets was higher when fragmentation had occurred during the first year of the mother's life. Whole-life seed production of plants from the seed bank was lower than seed production of uninjured plants (Table 3, Figure 2A).

**Table 2:** **Summary of fitted models for fitness characteristics obtained from experiment on ***Barbarea vulgaris*** and ***Barbarea stricta*****.

***B. vulgaris* + *B stricta***	***d.f*.**	***F*_ps_**	***p***	***B. vulgaris***	***d.f*.**	***F*_ps_**	***p***	***B. stricta***	***d.f*.**	***F*_ps_**	***p***
**A. NUMBER OF ALL SEEDS**											
Species (SP)	1	69.51	^***^	Regeneration type (REG)	1	31.86	^***^	Regeneration type (REG)	1	0.42	n.s.
Regeneration type (REG)	1	25.64	^***^	Ontogenic stage (O)	3	8.08	^***^	Ontogenic stage (O)	3	13.12	^***^
Ontogenic stage (O)	3	17.84	^***^	REGxO	2	3.39	^*^	REGxO	2	5.52	^**^
SPxREG	1	19.00	^***^								
SPxO	3	1.03	n.s.								
REGxO	2	3.99	^*^								
SPxREGxO	2	3.99	^*^								
**B. NUMBER OF VIABLE SEEDS**											
Species (SP)	1	131.19	^***^	Regeneration type (REG)	1	22.44	^***^	Regeneration type (REG)	1	1.87	n.s.
Regeneration type (REG)	1	12.48	^***^	Ontogenic stage (O)	3	11.27	^***^	Ontogenic stage (O)	3	5.17	^***^
Ontogenic stage (O)	3	10.76	^***^	REGxO	2	6.48	^**^	REGxO	2	2.70	n.s.
SPxREG	1	23.04	^***^								
SPxO	3	8.67	^***^								
REGxO	2	2.58	n.s.								
SPxREGxO	2	8.62	^***^								
**C. RATIO VIABLE/ALL SEEDS**											
Species (SP)	1	434.64	^***^	Regeneration type (REG)	1	2.78	n.s.	Regeneration type (REG)	1	40.845	^***^
Regeneration type (REG)	1	9.79	^**^	Ontogenic stage (O)	3	0.87	n.s.	Ontogenic stage (O)	3	12.115	^***^
Ontogenic stage (O)	3	7.34	^***^	REGxO	2	0.36	n.s.	REGxO	2	0.8219	n.s.
SPxREG	1	31.01	^***^								
SPxO	3	5.02	^**^								
REGxO	2	0.78	n.s.								
SPxREGxO	2	0.36	n.s.								
**D. ANNUAL IMMEDIATE REPRODUCTION**											
Species (SP)	1	166.40	^***^	Regeneration type (REG)	1	0.27	n.s.	Regeneration type (REG)	1	54.63	^***^
Regeneration type (REG)	1	10.77	^***^	Ontogenic stage (O)	3	11.61	^***^	Ontogenic stage (O)	3	29.95	^***^
Ontogenic stage (O)	3	21.81	^***^	REGxO	2	1.84	n.s.	REGxO	2	26.89	^***^
SPxREG	1	5.41	^*^								
SPxO	3	6.38	^***^								
REGxO	2	9.56	^***^								
SPxREGxO	2	1.28	n.s.								

*In the first column, both species are tested together; in the second and third columns, B. vulgaris and B. stricta, respectively, are tested separately. Effects of regeneration type—enforced ramets, seed bank, and control, as well as ontogenetic stage and species were tested on the following characteristics: **(A)** Number of all seeds **(B)** Number of viable seeds **(C)** Ratio viable/all seeds **(D)** Annual immediate reproduction. Degrees of freedom (d.f.), pseudo-F values (F_ps)_ and significance range are shown*.

* *p < 0.05*;

** *p < 0.01*,

*** *p < 0.00, n.s.—non-significant. Error d.f.: full model with both species = 296; partial model B. vulgaris = 151, partial model B. stricta = 145*.

**Table 3:** **Results of pair-wise tests of fitness characteristics for individual regeneration types and ontogenic stages: (A) Number of all seeds; (B) Number of viable seeds; (C) Ratio viable/all seeds; and (D) Annual immediate reproduction ***B. vulgaris*** and ***B. stricta***, were tested separately**.

	***B. vulgaris***		***B. stricta***	
	***t*_ps_**	***p***	***t*_ps_**	***p***
**A. NUMBER OF ALL SEEDS**				
FRG R1 vs. S R1	5.20	^***^	1.57	n.s.
FRG R2 vs. S R2	3.21	^***^	2.17	^*^
FRG REP vs. S REP	1.87	n.s.	1.84	n.s.
FRG R1 vs. NO INJURY	3.37	^**^	2.08	^*^
FRG R2 vs. NO INJURY	0.54	n.s.	0.48	n.s.
FRG REP vs. NO INJURY	0.85	n.s.	6.14	^***^
S R1 vs. NO INJURY	1.82	n.s.	0.51	n.s.
S R2 vs. NO INJURY	3.45	^***^	4.60	^***^
S REP vs. NO INJURY	2.40	^*^	5.01	^***^
**B. NUMBER OF VIABLE SEEDS**				
FRG R1 vs. S R1	4.79	^***^	1.96	n.s.
FRG R2 vs. S R2	1.16	n.s.	0.74	n.s.
FRG REP vs. S REP	1.81	n.s.	1.94	n.s.
FRG R1 vs. NO INJURY	4.07	^***^	1.30	n.s.
FRG R2 vs. NO INJURY	2.13	^*^	1.28	n.s.
FRG REP vs. NO INJURY	0.19	n.s.	2.37	^*^
S R1 vs. NO INJURY	1.63	n.s.	3.51	^***^
S R2 vs. NO INJURY	3.07	^**^	0.50	n.s.
S REP vs. NO INJURY	1.90	n.s.	0.05	n.s.
**C. RATIO VIABLE/ALL SEEDS**				
FRG R1 vs. S R1	0.36	n.s.	4.76	^***^
FRG R2 vs. S R2	1.38	n.s.	3.31	^**^
FRG REP vs. S REP	0.95	n.s.	2.92	^*^
FRG R1 vs. NO INJURY	1.17	n.s.	0.88	n.s.
FRG R2 vs. NO INJURY	0.74	n.s.	3.08	^**^
FRG REP vs. NO INJURY	0.12	n.s.	3.78	^***^
S R1 vs. NO INJURY	0.57	n.s.	5.37	^***^
S R2 vs. NO INJURY	1.12	n.s.	0.41	n.s.
S REP vs. NO INJURY	1.23	n.s.	0.87	n.s.
**D. ANNUAL IMMEDIATE REPRODUCTION**				
FRG R1 vs. S R1	1.66	n.s.	6.79	^***^
FRG R2 vs. S R2	1.14	n.s.	0.84	n.s.
FRG REP vs. S REP	0.67	n.s.	2.80	^*^
FRG R1 vs. NO INJURY	0.24	n.s.	0.16	n.s.
FRG R2 vs. NO INJURY	3.03	^**^	0.84	n.s.
FRG REP vs. NO INJURY	3.45	^***^	3.20	^***^
S R1 vs. NO INJURY	1.01	n.s.	6.16	^***^
S R2 vs. NO INJURY	2.29	^*^	0.10	n.s.
S REP vs. NO INJURY	2.16	^*^	0.44	n.s.

*Degrees of freedom (d.f.), pseudo t-test values (t_ps)_ and significance range are shown*.

* *p < 0.05*;

** *p < 0.01*,

*** *p < 0.00, n.s.—non-significant. FRG, enforced ramets; S, seed bank; R1, seeds sowed or roots of mother plant fragmented in first-year rosette phase; R2, seeds sowed or roots of mother plant fragmented in second-year rosette phase. REP, seeds sowed or roots of mother plant fragmented in reproductive phase. NO INJURY, control unfragmented plants. Full descriptions of regeneration types and ontogenetic phases (and their abbreviations) are in Table 1*.

In *B. stricta*, whole-life seed production of enforced ramets was higher than seed production of plants from the seed bank if fragmentation had occurred during the second-year vegetative phase of the mother plants (Table 3, Figure 2A). Seed production of enforced ramets and plants from the simulated seed bank were in most cases lower than seed production of control unfragmented plants (Table 3, Figure 2A).

### Number of viable seeds

If only viable seeds are taken into account, whole-life seed production was significantly influenced by the tested factors and their interactions (Table 2), similarly to production of all seeds. In *B. vulgaris*, seed production of enforced ramets was higher than seed production of plants from the seed bank if fragmentation had occurred during the first year of the mother's life. The same relationship is found when comparing seed production of enforced ramets and uninjured plants (Table 3, Figure 2B).

In *B. stricta*, seed production of enforced ramets was equal to control plants and also to plants from the simulated seed bank in most cases (Table 3, Figure 2B). Seed production of plants from the seed bank was significantly higher than seed production of uninjured plants if fragmentation had occurred during the first year of the mother's life (Table 3, Figure 2B).

### Ratio viable/all seeds

In *B. vulgaris*, the ratio of viable seeds to all seeds did not differed among regeneration types or ontogenetic stages (Table 3, Figure 2C). On the other hand, in *B. stricta*, the ratio was significantly higher in plants from the seed bank than in enforced ramets and also, in the case of fragmentation during the first year of the mother's life, than in uninjured plants (Table 3, Figure 2C). In enforced ramets, the ratio decreased with the ontogeny of the mother plant (Figure 2C).

### Annual immediate reproduction

Annual immediate reproduction was significantly influenced by the tested factors and their interactions in both species (Table 2). In *B. vulgaris*, reproduction of enforced ramets and plants from the seed bank did not differ (Table 3, Figure 2D), but decreased if fragmentation occurred during the second year of the mother's life.

In *B. stricta*, annual immediate reproduction was lower in enforced ramets than reproduction of plants from the seed bank and did not differ from reproduction of uninjured plants. In enforced ramets, annual immediate reproduction decreased if fragmentation had occurred during the reproductive phase of the mother plant (Table 3, Figure 2D). Plants from parallel sowing showed the highest annual immediate reproduction in the treatment that simulated their germination from the seed bank during the first year of the mother's life (Figure 2D).

## Discussion

In *B. vulgaris*, enforced ramets showed higher fitness than uninjured plants if their mothers were fragmented early in their ontogeny, and also higher fitness than plants originating in the seed bank. In *B. stricta*, enforced ramet fitness was lower than or similar to fitness of unfragmented plants and plants grown from seed. Fragmentation of the plant body therefore showed adaptive value, however the importance of enforced clonality reflected the individual species' habitat preferences. In particular, although the habitat of *B. stricta* does experience natural disturbances, it is not as frequently or severely disturbed as the man-made habitat of *B. vulgaris*, with the latter species therefore experiencing root system fragmentation more often. Our finding of increase in fitness by body fragmentation supports the idea of enforced clonality as a significant strategy in disturbed habitats.

### Enforced ramets

In our study, we used two enforced ramets from each mother to compare their fitness with that of one unfragmented individual or of one plant established from the seed bank. This setup affected our results because disturbance may lead to an even higher degree of fragmentation or, on the other hand, results only in injury to the plant body without causing fragmentation. Simple removal of aboveground biomass does not increase fitness of *B. vulgaris* (Martínková et al., 2016) in comparison with unfragmented plants; however, as we have shown here, formation of two enforced ramets of root origin significantly increases fitness. It is obvious that the advantage of enforced clonality is thus related to the type and also the severity of the particular disturbance. Thus, to maximize the gain from enforced clonality it is necessary to encounter appropriate disturbance severity. Indeed, if disturbance results in smaller fragments, their successful establishment and fitness may be reduced as it is dependent on stored reserves (Leakey et al., 1977; Klimešová and Klimeš, 2007). Species possessing enforced clonality are therefore probably preferring certain disturbance regime in order to encounter disturbance severe enough to increase their fitness but not too severe to kill them.

Higher fitness of enforced ramets is caused by removal of apical dominance during fragmentation, leading to production of numerous flowering shoots on fragments (see also Martínková et al., 2016). However, these shoots are usually shorter and less branched than uninjured shoots (Bartušková and Klimešová, 2010). Even though disturbance usually also severely affects competitors, if disturbance injures only some individuals within a community, their lower height and smaller root system might disadvantage them in competition. Unfortunately, competition was not simulated in our experiment. Nevertheless, the effect of competition on enforced clonality is not probably so strong since enforced clonality is disturbance-dependent and disturbance results in reduced competitive pressure (Wilson and Tilman, 1993). Another possible problem of enforced ramets may be delayed ontogeny and thus the inability to successfully set seeds during the year of fragmentation and postponement of reproduction to subsequent ones (Huhta et al., 2009; Piippo et al., 2009; Martínková et al., 2016). However, postponement of reproduction seems disadvantageous only when we consider situation in which not all individuals are fragmented, since the relevant comparison is between enforced ramets and unfragmented plants. Unfragmented plants set seeds in current year while enforced ramets a year later. Enforced ramets would face a disadvantage if recurrent disturbance comes before they finish reproductive cycle as unfragmented plants have already finished theirs. If all individuals are fragmented and enforced ramets are compared with plants established from seeds, they have an advantage due to the higher amount of stored reserves for faster regrowth, and postponement of their reproduction does not play a role as ramets reproduce at the same time as the plants established from seeds. Based on consideration of these scenarios, we can see that significance of enforced clonality is probably higher in situations in which disturbance fragmentizes all rather than only some individuals within a community.

Besides fitness increase, another advantage of enforced clonality is the deceleration of senescence. Enforced ramets of *B. vulgaris* survived 1 year longer than uninjured plants (Martínková et al., 2016). Indeed, enforced clonality is able to rejuvenate plants by resetting the aging clock in *B. vulgaris* (Martínková et al., 2016). Moreover, even though the number of reproductive events was the same due to postponement of reproduction in enforced ramets, whole-life seed production was higher in enforced ramets than in unfragmented plants. Since both *Barbarea* species start to reproduce during the second year of life and they behaved as polycarpic species (Martínková et al., 2016), advantage of enforced clonality may be more pronounced in annuals. In annual species with enforced clonality such as *Rorippa palustris* (Klimešová et al., 2008), injury-induced prolongation of the vegetative phase from 1 to 2 years could lead to a higher amount of stored reserves for generative reproduction in comparison to uninjured plants which germinate, reproduce and die within 1 year. Thus, the significance of enforced clonality probably varies among life-history strategies.

### Seed bank

In our experiment, disturbance timing was designed in relation to the ontogeny of maternal plants, and this could result in less-than- ideal timing for germination from the seed bank. Although seeds of the two study species are able to germinate during the whole year, the usual time for seedling establishment is the spring (MacDonald and Cavers, 1991), so that plants attain maximal size in the first growing season and are thus well prepared for flowering the next year. The signal for flowering in these species is low temperature during winter, not size, which plays this role in other short-lived plants; additionally, the length of the first growing season is an important influence on the seed production (Collins, 1981; Galen and Stanton, 1991). Therefore, it is not surprising that in our experiment plants from the seedbank frequently had lower fitness than both enforced ramets and uninjured plants, since fragmentation and simulation of establishment from the seed bank were not done exactly at the beginning of growing season. The timing of disturbance and hence the timing of germination affects fitness output. Nevertheless, disturbance is unpredictable, especially those caused by humans, which could occur at any time during the year. Thus, the design of our experiment actually reflects reality, and suggests that indeed enforced clonality is a useful strategy in highly unpredictably disturbed habitats.

### Advantage of enforced clonality in establishing new populations

Enforced clonality as a regenerative mode has a clear advantage in the situation in which disturbance hits a population that has not yet formed a seed bank. Furthermore, enforced clonality is especially important for short-lived otherwise non-clonal plants establishing as pioneer species on a new substrate, on places where vegetation was destroyed or during invasive or other colonization processes. Pioneer species can have quite small populations, and enforced clonality can greatly reduce their vulnerability to disturbances that would otherwise wipe them out. Overcoming such bottlenecks may in fact be the reason for retaining enforced clonality even though it does not seem to be so advantageous compared to regeneration from the seed bank in some situations. This may hold true for *B. stricta*, which occurs on natural habitats where experiencing body fragmentation is much less probable than on the man-made disturbed habitats inhabited by *B. vulgaris* (Dvořák, 1992). Since buds for regrowth after fragmentation are either formed adventitiously on roots only after injury or are a standard part of plant ontogeny, enforced clonality does not incur any costs for species. Therefore, once they attain the ability of resprouting it can be further kept without expense.

Another important aspect of enforced clonality is the ability of short-lived, non-clonal plants to survive disturbance when germination from the seed bank is not possible or is less successful due to unfavorable germination conditions (Bewley, 1997). More generally, enforced clonality could serve as insurance for species with problematic germination. Furthermore, enforced clonality may also have ecological effects even when enforced ramets are not capable of setting seeds (e.g., due to lack of mates or pollinating vectors or to insufficient growing season length) as these ramets can still play important roles such as competing for resources and serving as a source of litter (e.g., *Reynoutria* taxa, Bímová et al., 2003).

In conclusion, enforced clonality can increase the fitness of some short-lived species and thus bring a life history advantage. It is advantageous in habitats where a seed bank has not yet been formed. Our results imply that enforced clonality should be taken into account when studying population dynamics and life strategies of short-lived species from disturbed habitats.

## Author contributions

JM as a first author substantially contributed to the conception and design of the work. She was responsible for the experiment, analyzed data, interpreted results and wrote the manuscript. JK substantially contributed to the conception and design of the work and also to interpretation of data. She critically revised work and did final approval of the manuscript.

### Conflict of interest statement

The authors declare that the research was conducted in the absence of any commercial or financial relationships that could be construed as a potential conflict of interest. The reviewer Bi-Cheng Dong and handling Editor Fei-Hai Yu declared their shared affiliation, and the handling Editor states that the process nevertheless met the standards of a fair and objective review.
